# Supplementary material for: Assessing the NLRP3 Inflammasome Activating Potential of a Large Panel of Micro- and Nanoplastics in THP-1 Cells
Source: Biomolecules. 2022 Aug 9;12(8):1095. doi: 10.3390/biom12081095 (PMC9406042; doi:10.3390/biom12081095)
Supplement: Supplementary file 1 [file biomolecules-12-01095-s001.zip › biomolecules-1789230-supplementary.pdf]

Article

Assessing the NLRP3 Inflammasome Activating Potential of a Large Panel of Micro- and Nanoplastics in THP-1 Cells

Mathias Busch [1], Gerrit Bredeck [1], Friedrich Waag [2], Khosrow Rahimi [3], Haribaskar Ramachandran [1], Tobias Bessel [2], Stephan Barcikowski [2], Andreas Herrmann [3], Andrea Rossi [1] and Roel P. F. Schins [1,*]

[1] IUF–Leibniz-Research Institute for Environmental Medicine, 40225 Duesseldorf, Germany
[2] Technical Chemistry I, Center for Nanointegration Duisburg-Essen (CENIDE), University of Duisburg-Essen, 45141 Essen, Germany
[3] DWI–Leibniz Institute for Interactive Materials, Institute of Technical and Macromolecular Chemistry, RWTH Aachen University, 52074 Aachen, Germany
* Correspondence: roel.schins@iuf-duesseldorf.de; Tel.: +49-2113389369

Additional Figures

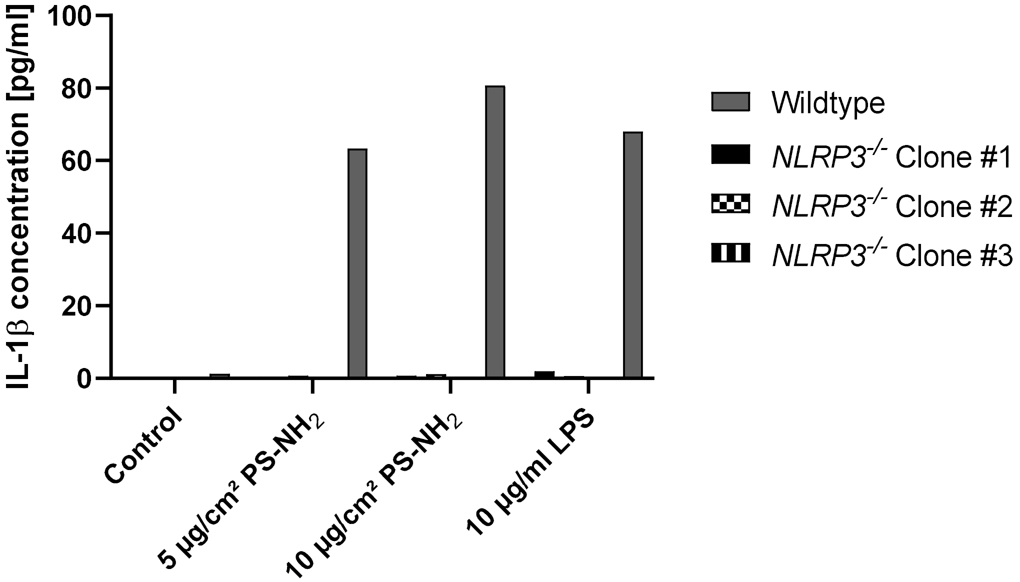

Figure S1. IL-1β release in THP-1 WT or *NLRP3*$^{-/-}$ cells after 24 h treatment with amino-modified polystyrene particles ($PS\text{-}NH_2$) or LPS. Cytokine release was assessed by ELISA. *N*=1.

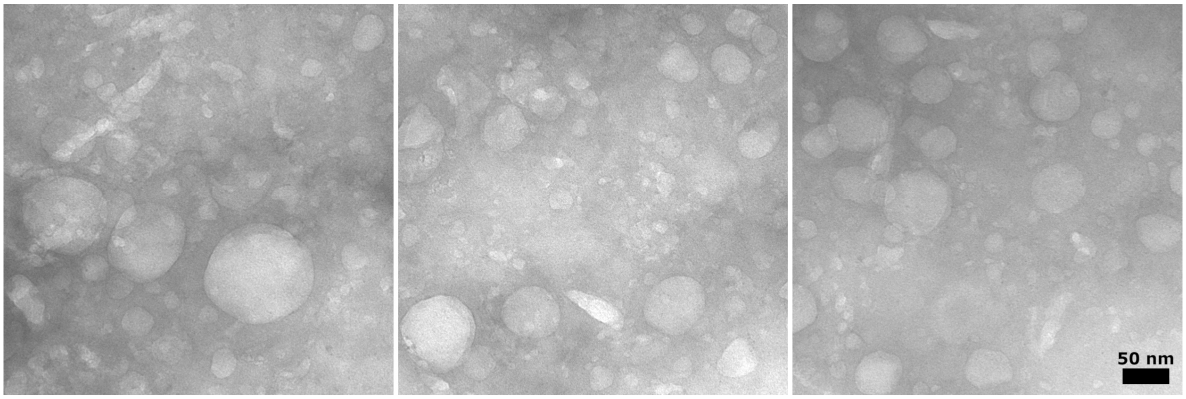

Figure S2. Exemplary TEM micrographs of the laser-generated PET nanoparticles. All micrographs have the same scale.

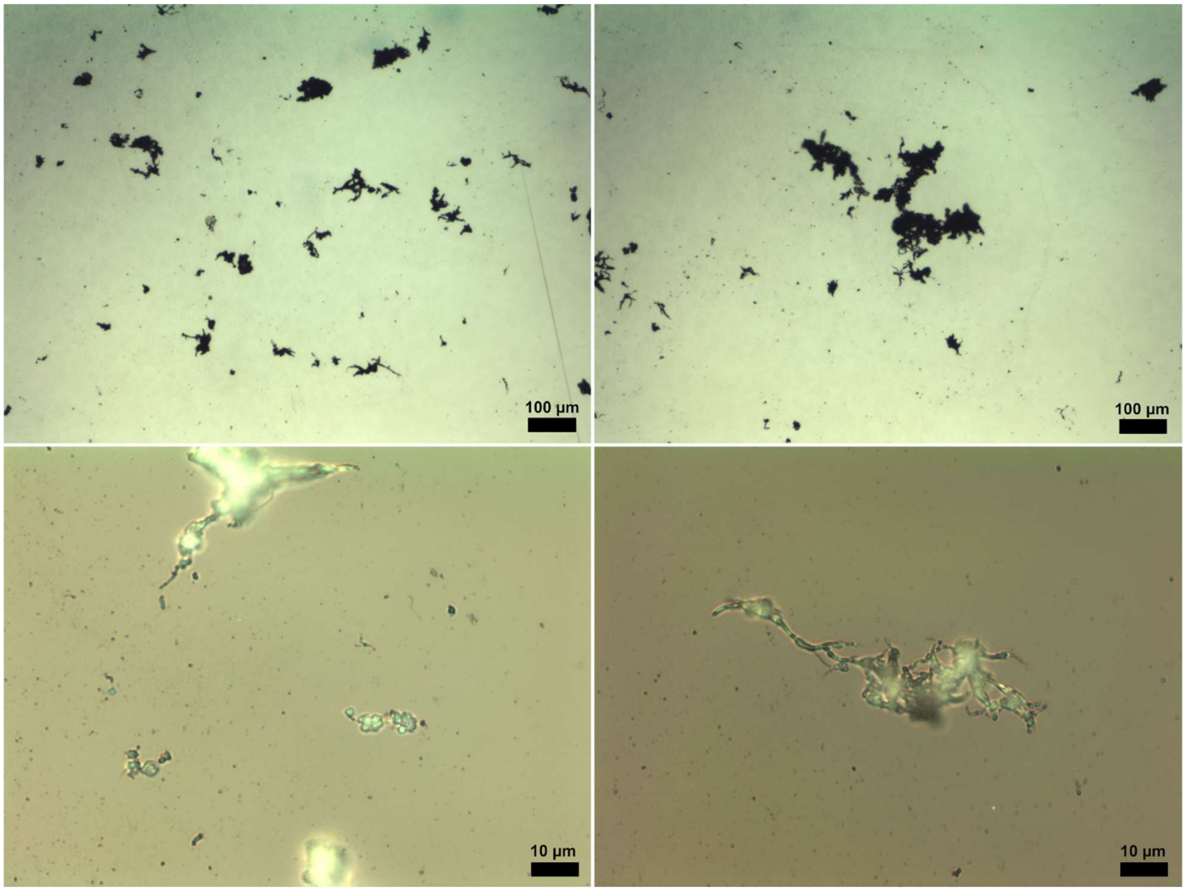

Figure S3. Exemplary light microscope images of the laser-generated PET microparticle fraction at 5x (top) and 50x (bottom) magnification.

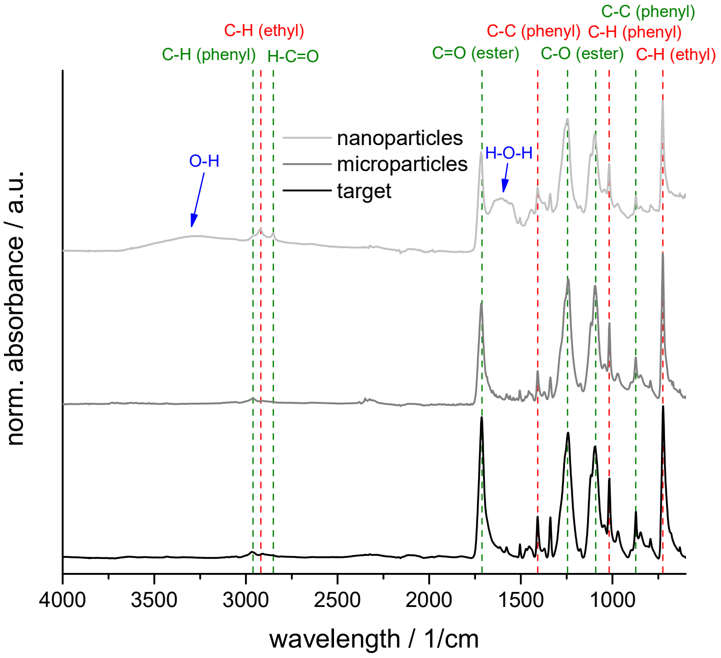

Figure S4. Normalized FT-IR absorbance spectra of the PET laser ablation target, the microparticle fraction, and the nanoparticle fraction. All spectra were normalized to the height of the peak of the C-H ethyl bond at 722 1/cm.

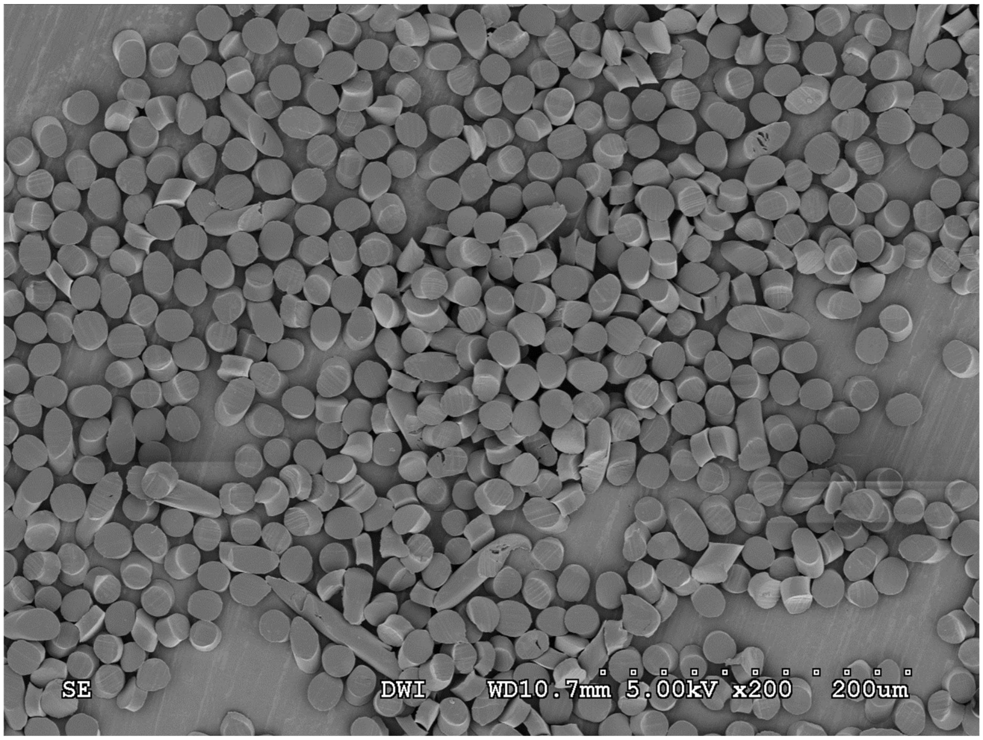

Figure S5. Exemplary SEM images of PES microfibers at 200x magnification.

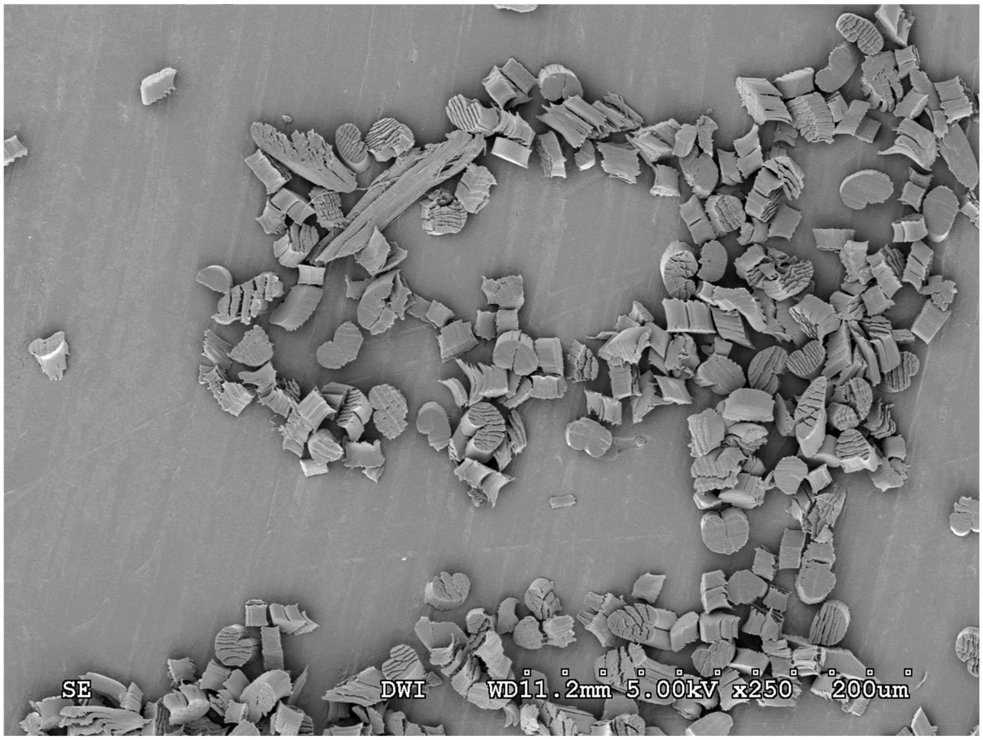

Figure S6. Exemplary SEM images of PAN microfibers at 250x magnification.

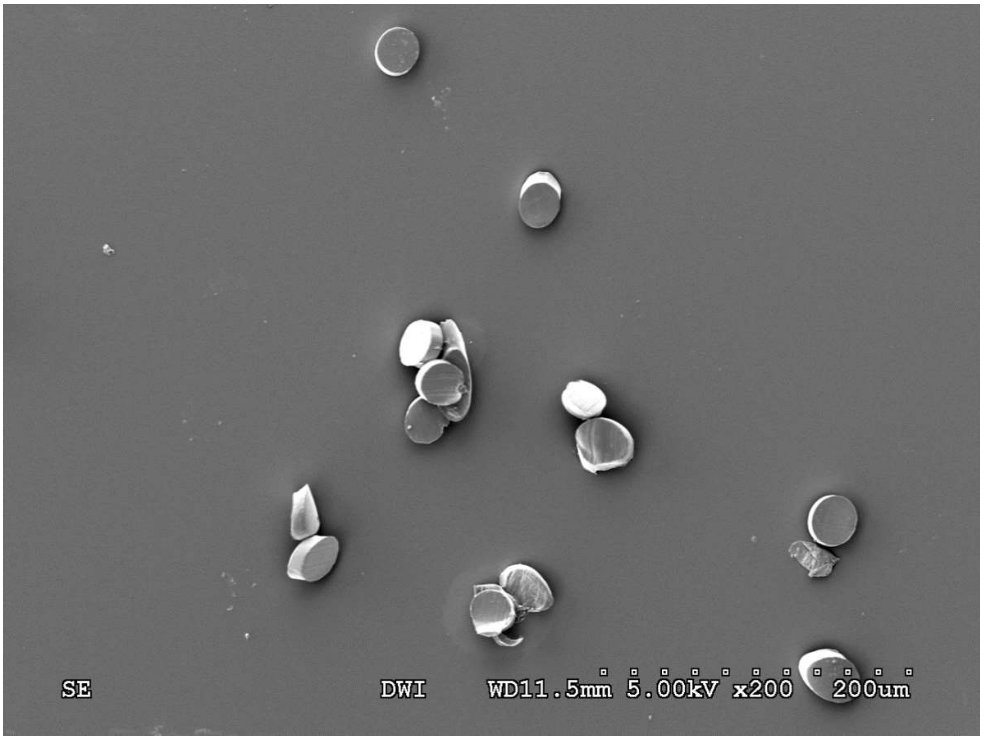

Figure S7. Exemplary SEM images of PA6 microfibers at 250x magnification.

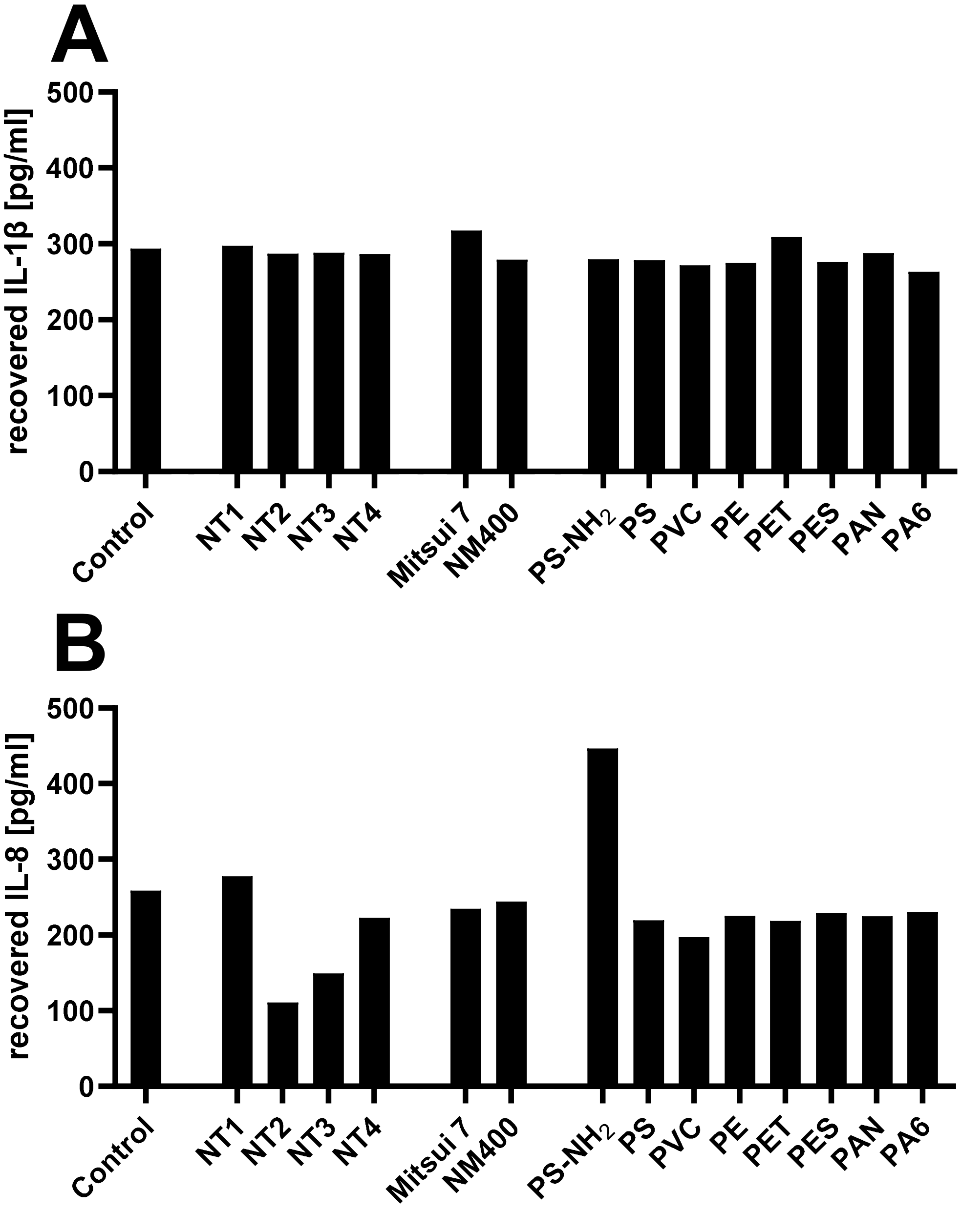

Figure S8. Recovered IL-1β (A) or IL-8 (B) after 24 h incubation of 500 pg/ml cytokine standard with 50 µg/cm² particle sample under experimental conditions, as assessed by ELISA. *N*=1.

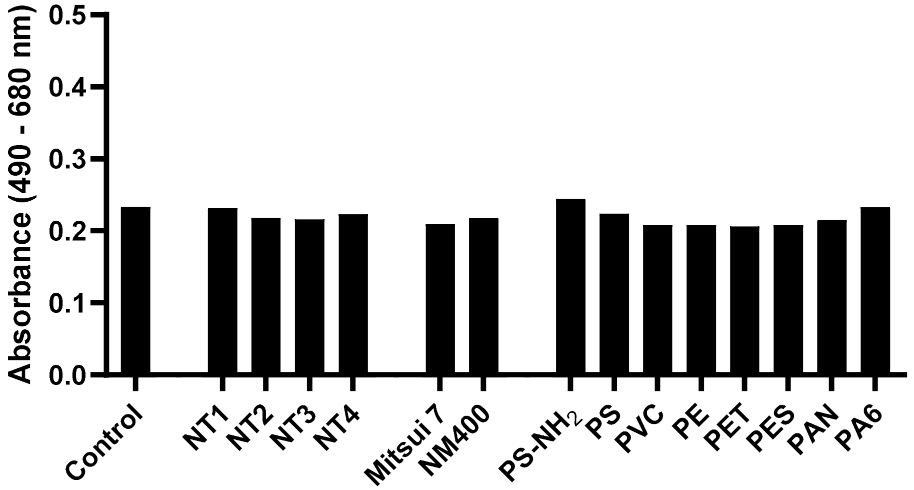

Figure S9. LDH activity in the supernatant after 24 h incubation of diluted cell lysate with 50 μg/cm² particle sample, as assessed by the LDH assay. *N*=1.
